# Ventilation strategies in burn intensive care: A retrospective observational study

**DOI:** 10.4103/2321-3868.126090

**Published:** 2014-01-26

**Authors:** Stefano Palazzo, Emma James-Veldsman, Caroline Wall, Michelle Hayes, Marcela Vizcaychipi

**Affiliations:** 1 Magill Department of Anaesthesia, Intensive Care Medicine and Pain Management, Chelsea and Westminster Hospital, London, UK; 2 Magill Department of Anaesthesia, Chelsea and Westminster Hospital, Fulham Road, London, SW10 9NH UK

**Keywords:** Burns, intensive care, ventilation, inhalation injury

## Abstract

Consensus regarding optimal burns intensive care (BICU) patient management is lacking. This study aimed to assess whether ventilation strategies, cardiovascular support and sedation in BICU patients have changed over time, and whether this affects outcome. A retrospective observational study comparing two 12-patient BICU cohorts (2005/06 and 2010/11) was undertaken. Demographic and admission characteristics, ventilation parameters, sedation, fluid resuscitation, cardiovascular support and outcome (length of stay, mortality) data were collected from patient notes. Data was analysed using T-tests, Fisher’s exact and Mann-Whitney U tests. In our study cohort groups were equivalent in demographic and admission parameters. There were equal ventilator-free days in the two cohorts 10 ± 12.7 vs. 13.3 ± 12.2 ventilator free days; *P* = 0.447). The 2005/06 cohort were mechanically ventilated more often than in 2010/11 cohort (568 ventilator days/1000 patient BICU days vs. 206 ventilator days/1000 patient BICU days; *P* = 0.001). The 2005/06 cohort were ventilated less commonly in tracheostomy group/endotracheal tube spontaneous (17.8% vs. 26%; *P* = 0.001) and volume-controlled modes (34.4% vs. 40.8%; *P* = 0.001). Patients in 2010/11 cohort were more heavily sedated (*P* = 0.001) with more long-acting sedative drug use (*P* = 0.001) than the 2005/06 cohort, fluid administration was equivalent. Patient outcome did not vary. Inhalational injury patients were ventilated in volume-controlled (44.5% vs. 28.1%; *P* = 0.001) and pressure-controlled modes (18.2% vs. 9.5%; *P* = 0.001) more frequently than those without. Outcome did not vary. This study showed there has been shift away from mechanical ventilation, with increased use of tracheostomy/tracheal tube airway spontaneous ventilation. Inhalation injury patients require more ventilatory support though patient outcomes do not differ. Prospective trials are required to establish which strategies confer benefit

## Introduction

Annually there are approximately 8000 hospital admissions due to burns in the United Kingdom (UK).[1] Many require intensive care unit (ICU) admission, with sedation, ventilation, and cardiovascular support forming an integral part of their management. However, a consensus regarding the most appropriate way to manage burn intensive care patients has yet to be reached.[2]

**Table:** 

Access this article online	
**Quick Response Code:**	**Website:** www.burnstrauma.com
	**DOI:** 10.4103/2321-3868.126090

We hypothesized that ventilation, sedation, and cardiovascular support strategies in ICU burn patients have changed with time, and differences correlate with mortality and morbidity outcomes. We also hypothesized that patients with inhalational injury are managed differently from those without. This retrospective observational study investigated these hypotheses.

## Materials and methods

### Clinical governance and data management

The study was retrospective and observational. In keeping with local guidelines, the research was considered and approved by the Clinical Governance office, Chelsea, and Westminster Hospital (reference number 509), rather than a research ethics committee. Data handling and confidentiality procedures were adhered to and Caldicott principles applied, as outlined in the Healthcare Quality Improvement Partnership Best Practice in Clinical Audit Guidance. Data were collected from patient notes between October and December 2011.

### Patient population

Data were collected on two cohorts of burn intensive care unit (BICU) patients. Cohort A included all patients admitted to the BICU in our regional burn centre between 01/05/05 and 30/04/06. Cohort B covered all patients admitted to the same unit between 01/05/10 and 30/04/11. Patients with thermal burns with total burn surface area (TBSA) > 15% admitted within 24 h of the burn incident were included.

### Demographic and outcome data

Demographic data such as patient age, gender, and weight were collected from the medical notes. TBSA estimation was taken from the initial assessment by the specialist burn surgery team. Diagnosis of inhalational injury was established from initial bronchoscopy findings, according to the Abbreviated Injury Score criteria.[3] Admission Acute Physiology and Chronic Health Evaluation II (APACHE II) score and Revised Trauma Scores were calculated from the medical notes and observation charts. Length of stay and outcome were obtained from the medical notes.

### Ventilation data

Data regarding ventilation mode and settings while on BICU were collected from nursing observation charts. These data are recorded hourly for the duration of the patient’s stay. Time periods where data were missing (e.g., patient in theatre) were excluded from analysis.

After data collection, ventilation mode was classified as one of four groups-own airway spontaneous, tracheostomy/tracheal tube spontaneous, volume-controlled ventilation, or pressure-controlled ventilation. The number of patient hours spent in each mode, the proportion of the total patient hours for each mode, and the number of ventilator free days at 28 days after admission to BICU was calculated.[4] Data regarding frequency of tracheostomy insertion and days of mechanical ventilation before tracheostomy insertion were also recorded.

### Sedation, fluid administration, and cardiovascular support data

Data regarding the type of sedative drug (short-acting (clonidine or propofol) or long-acting (midazolam or diazepam)) and level of sedation (measured using the Bloomsbury sedation score) in the first 72 h of BICU admission were collected from the nursing observation charts. The amount of intravenous fluid (ml/kg/%TBSA) administered in the first 72 h of admission, and the use of norepinephrine for cardiovascular support was also recorded.

### Clinical management

Given this was an observational retrospective study, management of patients was left at the discretion of the responsible clinical teams. The physicians involved in taking care of both cohorts of patients were the same.

The choice of mode of ventilation was made on an individual patient basis and influenced by the degree of patient injury, region of burn, frequency of theater trips, and level of sedation. The timing of tracheostomy was not protocolized and depended on the attending physician. Factors influencing the timing of tracheostomy included distribution and depth of burn and the availability of the surgical team to perform tracheostomy.

A local protocol guiding management of patients with inhalational injury was used during the study period. Early intubation with an uncut tracheal tube was performed in patients with clinical signs of upper airway injury. Chest X-ray was performed on admission, with bronchoscopy to confirm the diagnosis of inhalational injury at the earliest feasible opportunity, usually within 24 h of admission. Lung protective ventilation was used with FiO_2_ of 1.0 until carbon monoxide levels were less than 10%. Nebulized heparin 5000 IU 4-hourly, nebulized acetylcysteine 20% 4-hourly and nebulized salbutamol 2.5–5.0 mg 2-hourly were administered for 5–7 days in those with inhalational injury. Fluid administration was not automatically increased in response to the diagnosis of inhalational injury but based on clinical parameters suggesting fluid requirement and likely benefit. Target urine output was 0.5–1.0 ml/kg/h. A nasogastric or nasojejunal tube was inserted on admission and feeding started immediately. Chest physiotherapy with pulmonary toilet was performed at least 4-hourly. Prophylactic antibiotics or corticosteroids were not recommended, but daily sputum bacterial surveillance was performed and antibiotic administration guided by culture results and signs of infection. Patients were assessed daily for their readiness for extubation, with at least daily spontaneous breathing trials. Criteria determining readiness for extubation were: an ability to maintain the airway; respiratory rate <35; positive end expiratory pressure <0.5 kPa; assisted spontaneous breathing (ASB) pressure <1.0 kPa; minimal need for ventilatory support; good cough; no signs of delirium; and no need to be transferred to theater more than twice per week. In the earlier cohort, patients were weaned onto a T-piece for at least 2 h before extubation, while in the later cohort this practice was superseded by direct extubation if the patient had good tolerance to 0.5 kPa continuous positive airway pressure (CPAP).

### Statistical analysis

Statistical analysis was performed on Stata Statistical Software: Release 10 (College Station, TX: Stata Corp LP) using t-tests for variables with assumed normal distribution for which means were calculated (e.g., ventilator parameters), Fisher’s exact test for those variables that could be presented on contingency tables (e.g., gender, inhalation injury), and Mann-Whitney U tests for nonnormally distributed variables for which medians were calculated (e.g., age, weight).

## Results

### Patient population

A total of 24 patients were admitted to BICU during 2005/06 and 25 during 2010/11. Twenty five were subsequently excluded from analysis (12 in 2005/06 and 13 in 2010/11) as patients did not meet inclusion criteria or medical notes had been destroyed, leaving 12 patients in the 2005/06 cohort and 12 in the 2010/11 cohort.

### Demographics

The two patient groups were comparable in demographics [Table 1]. There was no significant difference in admission injury characteristics [Table 1]. The median admission APACHE II score was 25 in both groups, ranging from 8 to 37 in 2005/06 cohort and 14–35 in 2010/11 cohort (*P* = 0.772). Median revised trauma score on admission was 7.84 in both groups (2005/06 cohort, 5.97–7.84; 2010/11 cohort, 4.65–7.84; *P* = 0.940). Four (33.3%) patients had a tracheostomy inserted in 2005/06 cohort, 6 (50.0%) in 2010/11 cohort (*P* = 0.34). The mean (range) number of days of mechanical ventilation before tracheostomy insertion was 5.25 (3–9) in the 2005/06 cohort and 14 (3–26) in the 2010/11 cohort (*P* = 0.0833).

**Table 1:** Patient and injury characteristics and outcome in two BICU cohorts

	2005/06 cohort (*n* = 12)	2010/2011 cohort (*n* = 12)	*P*-value
Male	9/12 (75.0%)	7/12 (58.3%)	0.666*
Age (years)	49 (18–69)	39 (21–77)	0.260
Weight (kg)	72 (55–109)	75 (60–99)	0.794
% TBSA†	37.5 (15–75)	31.5 (15–90)	0.355
Inhalation injury	9/12 (75.0%)	5/12 (41.7%)	0.214*
Tracheostomy	4/12 (33.3%)	6/12 (50.0%)	0.340*
Trauma	1/12 (8.3%)	0/12	0.307
APACHE II	25 (8–37)	25 (14–35)	0.772
RTS‡	7.84 (5.97–7.84)	7.84 (4.56–7.84)	0.940
Length of BICU stay (days)	15 (1–37)	15 (2–54)	0.453
Mortality	7/12 (58.0%)	3/12 (25.0%)	0.214*

Values are median (range) or number (proportion): *P*-value (Mann-Whitney U or Fisher’s exact test*). APACHE II = Acute Physiology and Chronic Health Evaluation II score, BICU = Burn intensive care unit, ^†^TBSA = Total burn surface area, ^‡^RTS = Revised trauma score.

### Ventilation mode

**Figure 1:**
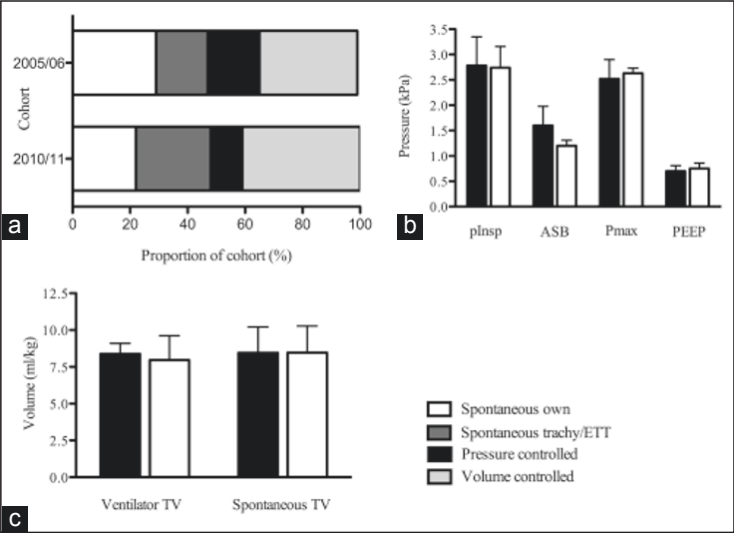
Graph comparing ventilation strategies and parameters in two time-separated burn intensive care unit cohorts. (a) Relative proportions of ventilation modes in burn intensive care unit (BICU) patients in 2005/06 and 2010/11 cohort. (b) Mean ventilator pressure parameters in two time-separated BICU cohorts. (c) Mean tidal volumes in two time-separated BICU cohorts in spontaneous and ventilator-controlled breathing. In b and c: Black bars, 2005/06 cohort; white bars, 2010/11 cohort. Error bars: 95% confidence interval.

Volume-controlled mechanical ventilation was the most common mode in both groups. However, a significant difference in time spent in each ventilation mode was found between the two cohorts [Figure 1a]. 2005/06 cohort ventilated more commonly in own-airway spontaneous (28.9% vs. 22.0%, *P* = 0.001) and pressure-controlled mechanical ventilation modes (18.1% vs. 11.*2%; P* = 0.001) than 2010/11 cohort. Conversely, 2010/11 cohort was more commonly ventilated in ETT/tracheostomy spontaneous (17.8% vs. 26.0%; *P* = 0.001) and volume-controlled ventilation modes (34.4% vs. 40.8%; *P* = 0.001).

### Ventilator days

In the 2005/06 cohort, the mean [standard deviation (SD)] ventilator free days at 28 days after admission was 10.0 (12.7), while it was 13.3 (12.2) in 2010/11 cohort (*P* = 0.447).

However, overall the 2005/06 cohort was mechanically ventilated more often than the 2010/11 cohort (568 ventilator days/1000 patient BICU days vs. 206 ventilator days/1000 patient BICU days; *P* = 0.001).

### Ventilation parameters

Ventilator parameters including ventilator and spontaneous tidal volume, inspiratory pressure, maximum airway pressure, and positive end-expiratory pressure (PEEP) did not vary significantly between the two groups [Table 2, Figure 1b and c]. Median-ASB pressure was significantly higher in 2005/06 cohort at 1.60 kPa than 2010/11 cohort at 1.20 kPa (*P* = 0.034).

**Table 2:** Comparison of ventilation parameters in two time-separated burn intensive care unit cohorts

Parameter	2005/06 cohort	2010/2011 cohort	*P*-value
Ventilator rate (breaths·min^−1^)	17.2 (15.1–19.4)	17.7 (16.0–19.3)	0.747
Spontaneous rate (breaths·min^−1^)	17.2 (12.2–22.2)	17.0 (13.0–21.0)	0.944
Ventilator tidal volume (ml/kg)	8.32 (7.67–9.10)	7.96 (6.30–9.62)	0.624
Spontaneous tidal volume (ml/kg)	8.45 (6.69–10.2)	8.45 (6.63–10.3)	0.995
Minute volume (L·min^−1^)	11.9 (9.28–14.1)	10.3 (8.17–12.5)	0.373
Inspiratory pressure* (kPa)	2.78 (2.21–3.35)	2.74 (2.31–3.16)	0.884
ASB† pressure (kPa)	1.60 (1.21–1.98)	1.20 (1.08–1.31)	0.034
Peak airway pressure* (kPa)	2.52 (2.14–2.90)	2.63 (2.00–2.73)	0.518
Positive end expiratory pressure (kPa)	0.70 (0.59–0.81)	0.75 (0.65–0.86)	0.479
Inspired O_2_ concentration (%)	46.9 (37.4–56.2)	40.2 (35.1–45.3)	0.184

Values are mean (95% confidence interval): *P*-value calculated with two-tailed t-test. *Inspiratory pressure applies during pressure controlled ventilation mode only. Peak airway pressure applies during volume controlled and spontaneous ventilation modes only. ^†^ASB = Assisted spontaneous breathing.

### Use of sedation

In the first 72 h of BICU admission, a sedation score between −1 and −3 (more heavily sedated) was recorded in 397 out of 599 total patient hours (66.3%) in the 2005/06 cohort, and 526 of 659 total patient hours (79.8%) in the 2010/11 cohort (*P* = 0.001). Conversely, a Bloomsbury score between 0 and 3 (less sedated/agitated) was recorded in 202/599 (33.7%) patient hours in the 2005/06 cohort, and 133/659 (20.2%) patient hours in the 2010/11 cohort (*P* = 0.001).

When used, long-acting sedative drugs were administered in 393/542 (72.5%) patient hours in the 2005/06 cohort, and 559/640 (87.3%) patient hours in the 2010/11 cohort (*P* = 0.001). Relatively more short-acting sedative drugs were used in 2005/06 cohort (149/542 (27.5%) patient hours) than 2010/11 cohort (81/640 (12.7%) patient hours), *P* = 0.001.

### Fluid administration and cardiovascular support

In the first 24 h of BICU admission, median (interquartile range) total fluid administration was 5.62 (3.53–6.74) ml/kg/%TBSA in 2005/06 cohort and 5.45 (3.41–8.62) ml/kg/%TBSA in 2010/11 cohort (*P* = 0.603). In the second 24 h period, in 2005/06 cohort 2.16 (1.56–4.43) ml/kg/%TBSA total fluid was administered, whilst in 2010/11 cohort 3.93 (2.82–4.47) ml/kg/%TBSA was given (*P* = 0.325). In the third 24 h period, patients in the 2005/06 cohort received 1.45 (0.94–2.60) ml/kg/%TBSA total fluid, while those in 2010/11 received 2.34 (0.69–3.69) ml/kg/%TBSA (*P* = 0.818).

In both cohorts, in the first 72 h of BICU admission the only inotrope or vasopressor used was norepinephrine. In 2005/06 cohort, norepinephrine was administered in 305 of 599 (50.9%) patient hours and it was administered in 373/659 (56.6%) patient hours in 2010/11 cohort (*P* = 0.0475).

### Outcome

Outcome measures were not significantly different between the two patient groups. Median length of BICU stay was 15 days in both groups (2005/06 cohort, 1–37 days; 2010/11 cohort, 2–54 days; *P* = 0.453). Mortality was higher in the 2005/06 cohort (7/12) than the 2010/11 cohort (3/12), though this did not reach statistical significance (*P* = 0.214).

### Inhalational injury

Fourteen patients had bronchoscopy-demonstrated inhalational injury, 9 in 2005/06 cohort and 5 in 2010/11 cohort. Patients from both cohorts with inhalational injury were compared with those without inhalational injury from 2005/06 and 2010/11. There was no significant difference in demographic parameters between patients with and without inhalational injury [Table 3]. Six patients (42.9%) with inhalational injury had a tracheostomy inserted, while 4 (40%) of those without inhalational injury were tracheostomied (*P* = 0.61).

**Table 3:** Patient and injury characteristics and outcome in BICU patients with and without inhalation injury

	No inhalation injury (*n* = 10)	Inhalation injury (*n* = 14)	*P*-value
Male	6/10 (60.0%)	10/14 (71.4%)	0.673*
Age (years)	38.0 (18–77)	46.5 (27–59)	0.660
Weight (kg)	68.0 (38–95)	73.5 (60–100)	0.252
% TBSA†	34.0 (15–90)	34.5 (15–75)	0.837
Tracheostomy	4/10 (40.0%)	6/14 (42.9%)	0.610*
Trauma	0/10	1/14 (7.1%)	1.000*
APACHE II	23.5 (8–35)	26 (19–37)	0.128
RTS^‡^	7.84 (7.10–7.84)	7.84 (4.65–7.84)	0.488
Length of BICU stay (days)	21.0 (3–54)	11.5 (1–42)	0.089
Mortality	3/10 (30%)	7/14 (50%)	0.421*

Values are median (range) or number (proportion): *P*-value (Mann-Whitney U or Fisher’s exact test*). BICU = burn intensive care unit, ^†^TBSA = Total burn surface area, APACHE II = Acute Physiology and Chronic Health Evaluation II, ^‡^RTS = Revised trauma score.

**Figure 2:**
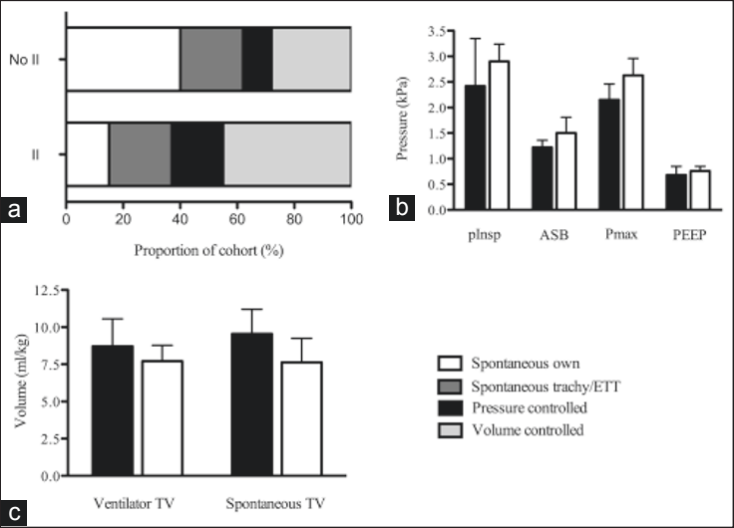
Graph comparing ventilation strategies and parameters in burns intensive care unit patients with and without inhalational injury. (a) Relative proportions of ventilation modes in burns intensive care unit (BICU) patients with and without inhalational injury (b) Mean ventilator pressure parameters in BICU patients with and without II. (c) Mean tidal volumes in BICU patients with and without II in spontaneous and ventilator-controlled breathing. In b and c: Black bars, without inhalation injury; white bars, inhalation injury. Error bars: 95% confidence interval.

In the inhalational injury group, the mean ventilator free days at 28 days after admission was 10.7 ± 11.8, while in those without inhalational injury there were 14.6 ± 13.4 ventilator free days (*P* = 0.478). Patients with inhalational injury were ventilated in volume-controlled (44.5% vs. 28.1%, *P* = 0.001) and pressure-controlled modes (18.2% vs. 9.5%, *P* = 0.001) more frequently than those without [Figure 2a]. Conversely, patients without inhalational injury were more likely to be breathing spontaneously without an airway device (15.4% vs. 39.9%, *P* = 0.001). There was no difference in the frequency of tracheal tube/tracheostomy airway spontaneous ventilation between the two groups (21.6% vs. 22.2%, *P* = 1.00).

Mean (95% confidence interval) peak airway pressure was significantly higher in the inhalational injury group; 2.63 (2.28–2.96) kPa than in patients without inhalational injury; 2.15 (1.84–2.46) kPa, *P* = 0.0475. However, there was no significant difference in all other ventilation parameters between the two groups [Table 4, Figure 2b and c].

**Table 4:** Comparison of ventilation parameters in burn patients with and without inhalation injury

Parameter	No inhalation injury	Inhalation injury	*P*-value
Ventilator rate (breaths·min^−1^)	17.0 (14.5–19.4)	17.8 (16.2–19.4)	0.513
Spontaneous rate (breaths·min^−1^)	19.3 (15.4–23.2)	15.6 (11.1–20.0)	0.203
Ventilator tidal volume (ml/kg)	8.71 (6.86–10.56)	7.81 (6.84–8.78)	0.304
Spontaneous tidal volume (ml/kg)	9.55 (7.87–11.2)	7.63 (6.02–9.24)	0.085
Minute volume (L·min^−1^)	10.5 (8.00–13.0)	11.3 (9.17–13.5)	0.597
Inspiratory pressure* (kPa)	2.42 (1.49–3.35)	2.90 (2.58–3.24)	0.118
ASB† pressure (kPa)	1.22 (1.06–1.36)	1.50 (1.19–1.81)	0.147
Peak airway pressure* (kPa)	2.15 (1.84–2.46)	2.63 (2.28–2.96)	0.048
Positive end expiratory pressure (kPa)	0.68 (0.55–0.85)	0.76 (0.67–0.85)	0.292
Inspired O_2_ concentration (%)	37.9 (31.4–44.4)	47.5 (40.2–54.9)	0.052

Values are mean (95% confidence interval): *P*-value calculated with two-tailed t-test. *Inspiratory pressure applies during pressure controlled ventilation mode only. Peak airway pressure applies during volume controlled and spontaneous ventilation modes only. ^†^ASB = Assisted spontaneous breathing.

No difference in total fluid administration in the first 72 h of BICU admission was seen between those patients with and without inhalational injury.

There was no significant difference in length of BICU stay or mortality between the two groups, though mortality was higher in those patients with inhalational injury (7/14) than without (3/10).

## Discussion

Consensus regarding the most appropriate way to ventilate BICU patients has yet to be reached.[2] Moreover, one of the major concerns in patients who are mechanically ventilated is the development of ventilator-induced lung injury.[5] This is attributed to shearing forces from delivery of high tidal volumes and excessive peak inspiratory pressures.[6] The use of lung-protective ventilation strategies with reduced tidal volumes has been shown to confer a mortality benefit in the acute respiratory distress syndrome (ARDS) population.[7,8] Extrapolating from this, burn patients are often ventilated with target tidal volumes of 6–8 ml/kg, though there is little evidence in this population.[6,9] Although ARDS is a recognized complication of severe burns,[10,11] particularly in patients with inhalational injury,[12] this is by no means universal. Other strategies that have shown some benefit in burn patients include high frequency percussive ventilation,[13,15] oscillation,[16,17] and avoidance of invasive ventilation altogether[18] though none have been universally adopted. Even the relatively elementary question of whether volume or pressure-controlled mechanical ventilation is most appropriate has yet to be resolved.[19,20] Therefore, this retrospective study aimed to establish whether ventilation strategies in BICU patients have changed over time and whether changes are associated with differences in outcome. It also investigated differences in sedation, fluid administration and cardiovascular support, and differences in ventilation between those with and without inhalational injury.

In this small cohort, there has been a trend toward increased spontaneous ventilation. Though there is no significant difference in the number of ventilator-free days at 28 days after BICU admission between the two cohorts, there were fewer ventilator-patient days in the 2010/11 patient group. Interestingly, this differs from previous reports of increased early mechanical ventilation amongst a Dutch burns cohort,[21] potentially due to regional variations. Though the number of patients breathing spontaneously without an artificial airway has fallen, there has been an increase in spontaneous ventilation with tracheostomy or tracheal tube. This is reflected in a greater proportion of patients having a tracheostomy inserted in the 2010/11 group, though this did not reach statistical significance. Intriguingly, the trend appeared to be toward later tracheostomy insertion in the 2010/11 cohort, which is in contrast to reports in the literature.[22,23]

The increase in spontaneous ventilation appears to be in spite of heavier sedation in the first 72 h of admission in the 2010/11 group. This may be attributable to the employment of “sedation holds” in the later cohort, or the use of heavier sedation early in admission, which, with better control of pain and wound healing,[24] is subsequently weaned allowing transition to spontaneous ventilation for the rest of the ICU stay. Either way, it is likely that differences in sedation do influence ventilation strategy.

We identified a trend toward decreased mortality in the 2010/11 patient group though this did not reach statistical significance due to small sample size. This was despite equivalent APACHE II and revised trauma score (RTS) scores, and could be attributed to differences in ventilation strategy. It is important to note, however, that fewer patients had sustained an inhalational injury in the 2010/11 group, and it is likely this contributes to the trends seen.

In mechanically ventilated patients, there has been an increase in the use of volume-controlled modes over time. Despite this, ventilation parameters when patients are mechanically ventilated have not changed. Importantly, there was no difference in tidal volumes delivered during mechanical ventilation. Furthermore, both groups were ventilated with mean tidal volumes at the upper limit of the recommended “lung-protective” value of 6–8 ml/kg. This might indicate awareness of the importance of low-volume ventilation strategies (hence an increase in volume-controlled ventilation) among clinicians, but continued concern regarding hypercapnia despite evidence it causes no harm.[25] Moreover, burns patients are different from other intensive care patients. The requirement for repeated surgical procedures results in multiple trips to theatre during an ICU stay. Mean tidal volumes delivered across the duration of an admission may, thus, be skewed by particularly high tidal volume delivery post-theatre. The combination of the burn itself (particularly if affecting chest wall compliance), aggressive initial fluid resuscitation causing relative fluid accumulation, and the inflammatory response to the burn may also make these patients more difficult to ventilate, leading to a requirement for higher tidal volumes. Number and duration of surgical procedures and burn location were not studied in our patient group, and may account for some of the differences in ventilation strategy seen. Our study has shown that high volumes of intravenous fluid continue to be given during the resuscitation phase of burns treatment. Seemingly identical strategies were used in 2005/06 and 2010/11, despite well-documented concerns around the dangers of excessive fluid administration and “fluid creep”[26] and the benefit of limited fluid resuscitation and permissive hypovolemia[27] in burned patients. The failure to change fluid administration approaches may contribute to the lack of mortality difference seen between the groups.

As would be expected, patients suffering from inhalational injury required increased mechanical ventilation with higher peak airway pressures than those without. This was matched by a trend toward higher ASB and inspiratory pressure in the inhalational injury group. This likely represents the ventilation settings required to achieve adequate gas exchange in patients with inhalation injury.

There was also a trend towards higher PEEP and lower tidal volumes in the inhalational injury group, though this did not reach statistical significance. This could represent a tendency towards high PEEP, low volume ventilation,[6] though may again be indicative of the settings required to maintain adequate oxygenation in patients with pulmonary perturbation. Given the sample size, it was not possible to correlate ventilation with outcome in those with inhalation injury.

The number of patients included limits the power of this study. It represents a relatively select population from the London and South-East England area. The data itself also relies on accurate recording by nursing staff, introducing an element of human error.

Despite these limitations, we have shown a shift away from mechanical ventilation, with an increase in the use of tracheostomy or tracheal tube spontaneous ventilation. This may be due to changes in the use of tracheostomies, though variations in inhalational injury rates between the two groups may also contribute. Increased spontaneous ventilation seems to be associated with a trend toward decreased mortality. There has been no change in ventilation set-up, indicating recommended lung-protective strategies are not being adopted uniformly in clinical practice. The advantages of this type of ventilation among BICU patients have yet to be established, however. Prospective, randomized clinical trials are required in this unique group of patients to establish whether particular approaches confer any benefit.
